# Interpreting artificial neural networks to detect genome-wide association signals for complex traits

**DOI:** 10.1093/nargab/lqag019

**Published:** 2026-02-23

**Authors:** Burak Yelmen, Maris Alver, Merve Nur Güler, Estonian Biobank Research Team, Flora Jay, Lili Milani

**Affiliations:** Estonian Genome Centre, Institute of Genomics, University of Tartu, Tartu, 51010,Estonia; Estonian Genome Centre, Institute of Genomics, University of Tartu, Tartu, 51010,Estonia; Estonian Genome Centre, Institute of Genomics, University of Tartu, Tartu, 51010,Estonia; Estonian Genome Centre, Institute of Genomics, University of Tartu, Tartu, 51010,Estonia; CNRS, INRIA, LISN, Paris-Saclay University, Orsay, 91190,France; Estonian Genome Centre, Institute of Genomics, University of Tartu, Tartu, 51010,Estonia

## Abstract

Investigating the genetic architecture of complex diseases is challenging due to the multifactorial interplay of genomic and environmental influences. Although GWAS have identified thousands of variants for multiple complex traits, conventional statistical approaches can be limited by simplified assumptions such as linearity and lack of epistasis. In this work, we trained artificial neural networks using genome-wide genotype data to predict simulated and real complex traits. We extracted feature importance scores via different post hoc interpretability methods to identify potentially associated locus/loci (PAL) for the target phenotype and devised an approach for estimating *P*-values for the detected PAL. Simulations demonstrated that associated loci can be detected with good precision using strict selection criteria. By applying our approach to the schizophrenia cohort in the Estonian Biobank, we detected multiple loci not identified by linear methods. There was significant concordance between PAL and loci previously associated with schizophrenia and bipolar disorder, with enrichment analyses of genes within the identified PAL predominantly highlighting terms related to brain morphology and function. With advancements in model optimization and uncertainty quantification, artificial neural networks have the potential to enhance the identification of genomic loci associated with complex diseases, offering a more comprehensive approach for GWAS.

## Introduction

Understanding the genetic component of complex diseases is challenging due to high polygenicity and the influence of confounding factors. Genome-wide association studies (GWAS) have been crucial for the discovery of thousands of disease-related genomic loci in the past two decades [1], and they continue to be relevant due to ever-expanding biobanks with extensive phenotype and genotype data, which enable the detection of the most minuscule but statistically significant signals. Despite the immense success and broad applications across various complex traits, conventional GWAS methodology still relies on linear models (i.e. logistic and linear regression) for detecting signals. More recently, linear mixed models have been utilized to incorporate random and fixed effects, primarily to account for relatedness and population structure without data pruning [2, 3]. While these advancements have generally improved GWAS outcomes, they do not diverge from the core assumptions of linearity and additivity in genetic models for complex traits. A natural alternative for nonlinear modelling of such traits would be machine learning, and particularly deep learning, since sufficiently large neural networks can approximate any bounded continuous function as defined in the universal approximation theorem [4]. Hence, not surprisingly, various models have been developed for the prediction of disease status from genetic data following the success of deep learning applications in many domains [5, 6]. Despite the state-of-the-art performance in prediction, a main drawback of employing such complex architectures is the inherent difficulty of interpretability due to the high number of parameters at various abstraction levels. In this context, the development and integration of interpretability methodology is not only important for understanding model biases and improving architectures but also for detecting novel features (e.g. genomic loci, haplotypes, and specific genetic variants) associated with the target phenotype.

Various approaches have been proposed to tackle interpretability for the discovery of trait-associated loci, ranging from intrinsically interpretable architectures based on domain knowledge, such as functional gene annotations [7–9], to post hoc analysis of trained models [10–14]. Despite these important advancements, multiple challenges remain for the widespread use of interpretable deep learning in genomics. Many of the proposed methods rely on either specific architectures, specific interpretability methods, or *a priori* biological knowledge. Furthermore, most of these methods require an initial feature-selection step to reduce computational complexity. For instance, GenNet architecture is designed to cluster single-nucleotide polymorphisms (SNPs) into genes and genes into clusters, relinquishing full connectivity of neural networks. This is powerful for biological interpretability but potentially restrictive for complex traits with not-fully-understood mechanisms, as the authors also report that a randomly connected network outperforms designs with prior knowledge for schizophrenia [7]. Another example is DeepCOMBI framework, in which a neural network is trained for phenotype prediction and layer-wise relevance propagation is used to select relevant SNPs [12]. Despite good results, the method is restricted to single chromosomes and requires manual optimization of multiple steps for different datasets. Furthermore, the neural network is utilized only to select the top *k* SNPs, for which a regular ${\chi }^2$ is performed to obtain significance, which means that SNPs are eventually tested separately. As a final example, DeepWAS framework uses a pre-trained neural network to obtain functional annotations for SNPs, which are grouped based on these annotations and tested with multivariate association analysis [15]. This is an interesting concept, improving interpretability via switching from conventional post hoc to pre hoc functional annotation, but it focuses on regulatory SNPs as it relies on the initial grouping based on the pre-trained neural network.

In this work, we (i) propose a score- and model-agnostic framework for identifying potentially associated locus/loci (PAL) from neural network models trained for phenotype prediction using genome-wide SNP array data, (ii) introduce an approach for obtaining *P*-values for the detected PAL, (iii) compare the performance of various post hoc and model-agnostic interpretability methods using simulations, and (iv) apply our approach first to a small preliminary type 1 diabetes (T1D) cohort and then to the schizophrenia (SCZ) cohort from the Estonian Biobank (EstBB) [16, 17]. SCZ is a highly complex and polygenic psychiatric disorder with ${\sim}60\% \hbox{-}80\%$ heritability estimate from twin studies, whereas SNP heritability is ${\sim} 25\%$, and present genomic approaches potentially only explain ${\sim} 40\%$ of trait variability [18]. Despite many detected associations in recent large-scale GWAS [19, 20], the genetic mechanisms of SCZ are still poorly understood, and small cohort sizes were shown to be insufficient to detect significant associations [21]. Considering all these challenges, after testing our method on a preliminary small T1D dataset (due to the established *HLA* region SNPs with a very strong association signal [22]), we used the SCZ cohort to demonstrate our method’s utility for GWAS.

## Materials and methods

### Data

We assembled the SCZ cohort from the EstBB dataset so that all patients (ICD codes F20–F29) would have at least one antipsychotic prescription and have their first diagnosis after 2006 with the diagnosis age between 14 and 60 [23]. As controls for the SCZ cases, we first assembled a set of individuals without any mental, behavioural, and neurodevelopmental disorders (no ICD F* diagnosis) and no record of antipsychotic prescriptions. From this filtered set, controls were selected to match the age, sex, and BMI distribution of the case cohort with four controls per case using MatchIt [24]. We used exact matching (method = ‘exact’) for sex and nearest neighbour matching (method = ‘nearest’) for BMI and age, resulting in 1814 cases (642 male, 1172 female) and 7325 controls (2586 male, 4739 female). In addition, the whole dataset was pruned to eliminate relatedness based on identity by descent (pi_hat < 0.2) [25, 26]. We curated the genotype data of the EstBB SCZ case/control cohort derived from the GSA array (Illumina’s GSA-MD-24v1, GSA-MD-24v2, ESTchip1_GSA-MD-24v2, ESTchip2_GSA-MD-24v3, 2022-09-14 snapshot). Genotype data quality control was performed according to best practices. Specifically, individuals with call rate ${\lt} 95\%$, who deviated ± 3SD from the samples’ heterozygosity rate mean or showed mismatch between heterozygosity of the X chromosome and sex based on phenotype data were excluded; all AT and GC SNPs, invariable SNPs, SNPs showing potential traces of batch bias, poor cluster separation results, and inconsistent allele frequency among any of the EstBB genotyping experiments were removed. The final set contained 290 522 SNPs across 22 autosomal chromosomes after further removing SNPs unique to at least one version of the array, indels, and filtering for bi-allelic regions. Using the same array framework, we additionally compiled a T1D cohort consisting of 552 case and 1104 control samples with 20 714 SNPs only from chromosome 6 for preliminary analysis.

### Simulations

We obtained simulated phenotypes from the same combined SCZ case/control genotype dataset. Instead of simulating genotypes, we simulated phenotypes to keep genome distribution as realistic as possible. In more detail, let $X$ represent the genotype matrix where $X_{ij}$ is the genotype at position $j$ for an individual $i$ with possible values −1 (AA), 0 (AB), and 1 (BB); $\beta$ the vector of genetic weights sampled from the standard normal distribution $N$(0,1); $\epsilon _i$ the noise term sampled from $N$(0, $k \sigma ^2$), where $k$ is a scaling factor and $\sigma ^2$ is the variance of the total genetic effects. Then, the continuous phenotype $Y$ for individual $i$ is defined as


\begin{eqnarray*}
Y_i &=& \sum _{j \in D} f^D_j(X_{ij}) + \sum _{j \in R} f^R_j(X_{ij}) + \sum _{(j,k) \in I} f_{jk}(X_{ij}, X_{ik}) \\&+& \sum _{(j,k,l) \in I} f_{jkl}(X_{ij}, X_{ik}, X_{il}) + \epsilon _i,
\end{eqnarray*}

where the initial four terms define the genetic effects based on dominant, recessive, two-way (between two SNPs), and three-way (between three SNPs) interactions; $D$, $R$, and $I$ are disjoint sets of genomic positions for dominant, recessive, and interaction effects. For dominant and recessive effect positions, contribution to the phenotype is defined by functions $f_j^D$ and $f_j^R$:


\begin{eqnarray*}
f^D_j(X_{ij}) = \left\lbrace \begin{array}{@{}l@{\quad }l@{}}\beta _j \cdot X_{ij} & \text{if } X_{ij} \ne -1, \\0 & \mathrm{otherwise}. \end{array}\right.
\end{eqnarray*}

\begin{eqnarray*}
f^R_j(X_{ij}) = \left\lbrace \begin{array}{@{}l@{\quad }l@{}}\beta _j \cdot X_{ij} & \text{if } X_{ij} = \mathrm{1}, \\0 & \mathrm{otherwise}. \end{array}\right.
\end{eqnarray*}

Two-way and three-way interaction effects are defined by


\begin{eqnarray*}
f_{jk}(X_{ij}, X_{ik}) = \beta _{jk} \cdot X_{ij} \cdot X_{ik},
\end{eqnarray*}

\begin{eqnarray*}
f_{jkl}(X_{ij}, X_{ik}, X_{il}) = \beta _{jkl} \cdot X_{ij} \cdot X_{ik} \cdot X_{il}.
\end{eqnarray*}

Given a defined threshold $\tau$, the continuous phenotype is then binarized by


\begin{eqnarray*}
B_i = \left\lbrace \begin{array}{@{}l@{\quad }l@{}}1 & \text{if } Y_i > \tau, \\0 & \mathrm{otherwise}. \end{array}\right.
\end{eqnarray*}

In practice, we chose $\tau$ to have approximately the same number of cases (${\sim}$1814) as the real SCZ cohort. We simulated a total of six scenarios with different noise scaling factors ($k$ = 1, $k$ = 2, $k$ = 3) and two different numbers of causal SNPs (*n* = 100 and *n* = 1000, randomly chosen over the whole genome) where all four genetic effect types (dominant, recessive, two-way, and three-way interactives) had positions with ratios of 5:5:4:6, respectively (i.e. for *n* = 100: 25, 25, 20, 30 and for *n* = 1000: 250, 250, 200, 300), yielding equal numbers of causal SNPs for dominant/recessive versus interactive effect types. The two sets of SNPs (*n* = 100 and *n* = 1000) used to simulate phenotypes can be found in the Supplementary Table S1. Genotypes were represented by −1, 0, and 1 instead of 0, 1, and 2 to provide equal importance to either allele in a given position (considering that the neural network weights are initialized with Kaiming uniform distribution, including negative and positive values uniformly). Nevertheless, our preliminary trials with 0, 1, and 2 representations provided similar results in terms of model training.

### Neural network model

Models trained for binary trait classification were all feedforward fully connected neural networks with an input layer, two hidden layers, and an output layer. The specific architecture consists of an initial dropout layer to randomly mask a proportion of features (*P* = 0.99) at each batch training; a linear layer with input size = 290 522 (corresponding to all genome-wide SNPs) and output size = 290; a dropout layer (*P* = 0.6) followed by ReLU activation; a linear layer with input size = 290 and output size = 29; a dropout layer (*P* = 0.6) followed by GELU activation; and a linear layer with input size = 29 and output size = 1 followed by sigmoid activation function. This equates to a total of 84 260 139 trainable parameters. Training was performed using cross-entropy loss function and the Adam optimizer with learning rate = 1e−5 and weight decay = 1e−3 for regularization. Initially, we implemented a weighted loss function to address the class imbalance in the dataset; however, this approach was ineffective (i.e. none of the training runs showed a reduction in validation loss). As an alternative, we duplicated the case samples and reduced the control sample size by half through random subsampling to balance the case/control ratio [27]. We additionally added random noise $N$(0,0.1) both to class labels (case label = 1, control label = 0) and input genotypes to increase generalizability.

For the preliminary analysis with the small T1D dataset, the architecture consisted of an initial dropout layer to randomly mask a proportion of features (*P *= 0.50) at each batch training; a linear layer with input size = 20 714 (corresponding to SNPs in chromosome 6) and output size = 82; a dropout layer (*P* = 0.6) followed by ReLU activation; a linear layer with input size = 82 and output size = 8; a dropout layer (*P *= 0.6) followed by GELU activation; and a linear layer with input size = 8 and output size = 1 followed by sigmoid activation function. The intuition for this specific design was to keep the same number of active trainable parameters (i.e. total parameters that will be updated for each batch after dropout masking) as the previous models, since we used the same resolution with the same array framework for all training datasets. In other words, simulation, SCZ, and T1D models all had $\sim$510 000 parameters updated at each batch.

Full models for simulated and SCZ datasets were trained for 1000 epochs with batch size = 256 using 150 cases and 150 control samples as validation, whereas preliminary T1D model was trained for 100 epochs using 100 case and 100 control samples as validation. All models were coded with Python-3.9 and PyThorch-1.11 [28].

### Interpretability methods

While there is a plethora of different interpretability methods for deep learning models, we focused on three post hoc and model-agnostic approaches to be able to produce a general framework suitable for most architectures. Two of them, gradients w.r.t. input [saliency maps (SM)] [29] and integrated gradients (IG) [30], are based on gradients and applicable to any differentiable model. SM creation is a commonly used and straightforward approach where gradients w.r.t. input are obtained to assess which features in a given input are important for the output probability. For a binary classification neural network model $f$ and input vector $x$, the saliency (with absolute values) for the $j\mathrm{th}$ feature is defined as


\begin{eqnarray*}
{\mathrm{SM}}_j(x) = \left| \frac{\partial f(x)}{\partial x_j} \right|.
\end{eqnarray*}

IG is an improvement over the simpler gradient approach where gradients of the model’s output with respect to the input are integrated along a path from a baseline input to the actual input. For a neural network model $f$, input vector $x$, baseline *x′* , and scalar $\alpha \in [0, 1]$, integrated gradient for the $j\mathrm{th}$ feature is defined as


\begin{eqnarray*}
{\rm {IG}}_j(x) = (x_j - x^{\prime }_j) \int _{0}^{1} \frac{\partial f(x^{\prime } + \alpha (x - x^{\prime }))}{\partial x_j} \, d\alpha.
\end{eqnarray*}

As the authors of the method emphasize, the choice of baseline vector is important so that a meaningful interpolation can be achieved in the given domain. In our application, we tested various baselines and decided on using a vector of zeros (where the input is a case genotype), which, in preliminary analyses, provided better outcomes (i.e. lower FP and higher TP counts) compared with (i) (input = case genotypes) − (baseline = vector of average allele dosages for the whole dataset) and (ii) (input = case genotypes) − (baseline = control genotypes) settings (Supplementary Fig. S1). In this regard, our choice of genotype representations with -1, 0, and 1 might also have been helpful, since a vector of zeros would correspond with a completely heterozygous genome, without preference over any allele in a given position. We used the Captum-0.6.0 Python library [31] for calculating SM and IG.

Permutation (PM)-based feature importance is another model-agnostic technique (in this case, the model does not even need to be differentiable) that measures the impact of feature perturbations on model output [32]. For a neural network model $f$, input vector $x$ and perturbed input vector $x^{(j)}$ (where the $j\mathrm{ th}$ feature is replaced by a random feature), the permutation feature importance for the $j\mathrm{th}$ feature is defined as


\begin{eqnarray*}
{\mathrm{PM}}_j(x) = \left| f(x) - f(x^{(j)}) \right|.
\end{eqnarray*}

Here, we opted to measure the rate of change in predictions for the feature importance instead of a performance metric such as accuracy, as this approach increases sensitivity. This is because each feature is expected to have only a small contribution without causing substantial alteration in the output prediction.

Using these three methods, we obtained absolute feature importance scores for the case samples (both in real and simulated scenarios) for all trained models, applied L1 normalization across genomic positions, and averaged the scores over samples. We call this averaged value mean attribution score (MAS). L1 normalization was performed to transform the score of each feature for a given sample into a measure of contribution, enabling comparisons between samples.

### Feature importance to potentially associated loci

To account for the stochasticity of neural networks and reduce false positive rate, we devised a streamlined method for obtaining PAL from MAS computed from multiple models trained with different seeds. More specifically, let $A$ be the MAS matrix sized $m \times L,$ where $m$ is the number of models (i.e. models trained with different seeds) and $L$ is the length of the genotype. We obtain the average MAS vector $\mu$:


\begin{eqnarray*}
\mu = \left(\mu _1, \mu _2, \ldots , \mu _L\right),
\end{eqnarray*}

where


\begin{eqnarray*}
\mu _j = \frac{1}{m} \sum _{a=1}^m A_{aj} \quad \text{for } j = 1, 2, \ldots , L.
\end{eqnarray*}

Then, we set a threshold $\theta$ and obtain weights $w$ based on the number of occurrences above the $\theta$ threshold among $m$ models trained with different seeds:


\begin{eqnarray*}
w_j = \frac{1}{m} \sum _{a=1}^m \mathbf {1}(A_{aj} > \theta ), \quad \text{where } \mathbf {1} \text{ is the indicator function.}
\end{eqnarray*}

To obtain PAL, first we define the adjusted mean attribution score (AMAS) as


\begin{eqnarray*}
{\rm {AMAS}}_j = w_j \times \mu _j, \quad \text{for all } j \text{ such that } \mu _j > \theta.
\end{eqnarray*}

Then, the PAL set contains all positions above *θ* threshold based on their AMAS:


\begin{eqnarray*}
{\mathrm{PAL_{AMAS}}} = \left\lbrace j \mid {\rm {AMAS}}_{j} > \theta \right\rbrace.
\end{eqnarray*}

We can also define PAL as positions above *θ* threshold in all $m$ models:


\begin{eqnarray*}
{\rm {PAL_{Common}}} = \left\lbrace j \mid \forall a, A_{aj} > \theta \right\rbrace.
\end{eqnarray*}

**Figure 1.**
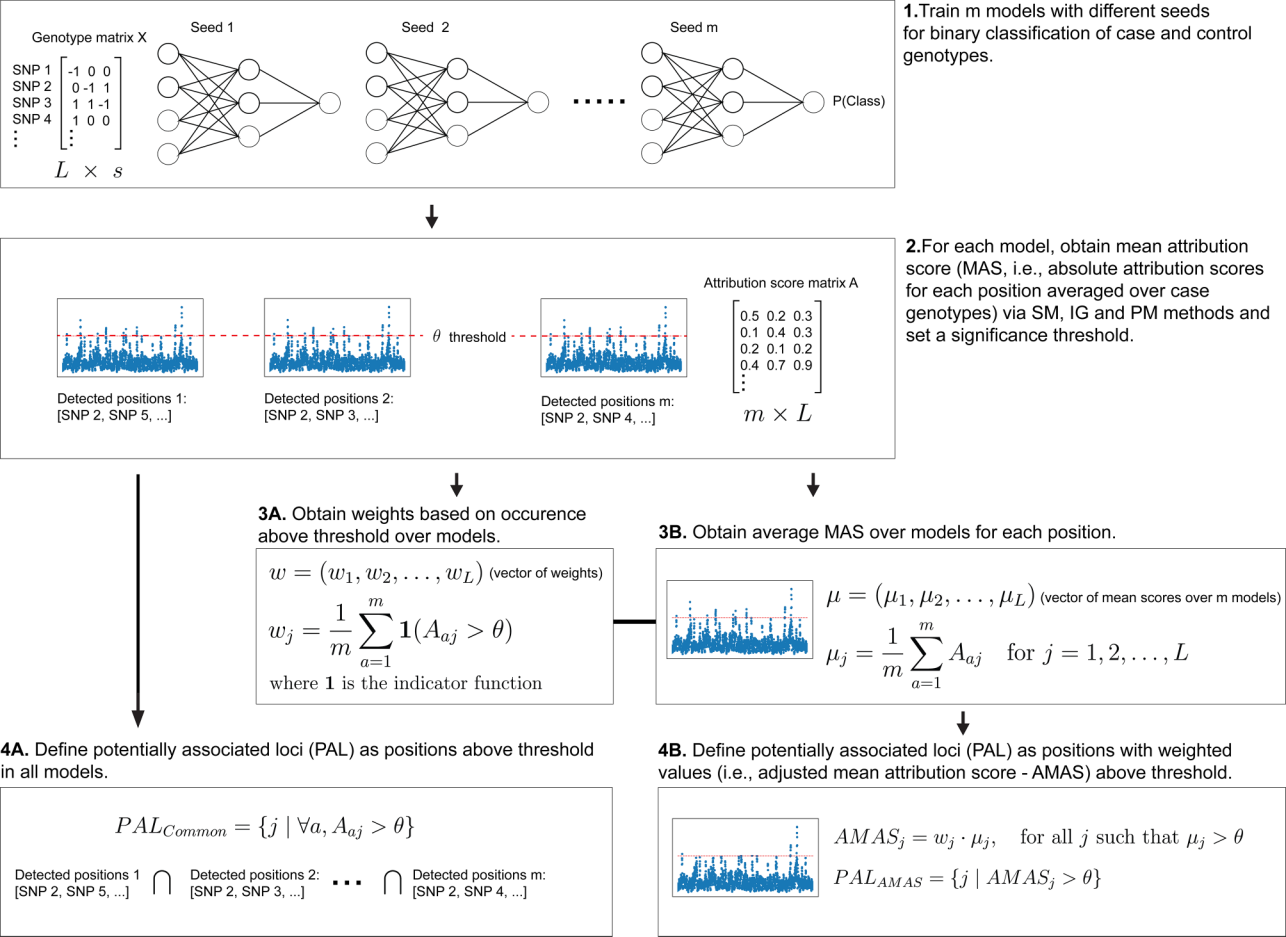
Overview of the approach for obtaining PAL from feature attribution scores obtained via SM, IG, and PM methods.

In practice, we chose $m=10$ for models trained with different seeds to be able to perform a large number of tests with realistic computational resources. Since multiple SNPs in high LD were detected by models trained with different seeds, we considered these positions ($r> 0.5$, absolute Pearson’s correlation coefficient calculated based on the whole case and control samples) across models as common signals to increase detection power. In other words, we clumped these SNPs detected by different models if they were in LD, and we did not perform other SNP-to-locus aggregation. Our choice of terminology (i.e. PAL, regardless of whether a signal consists of a single or a collection of SNPs) is to prevent implying that the detected SNPs are actual signals since models trained with different seeds tended to detect different SNPs in LD. Furthermore, we set the threshold $\theta$ to 99.99 percentile (strict) and 99.95 percentile (relaxed). Although the choice of $\theta$ is essentially arbitrary, the strict threshold would yield 29 and the relaxed threshold 145 SNPs based on MAS before weighting, which are plausible numbers for maintaining a low false positive rate, given the high polygenicity of schizophrenia [18]. An overview of our proposed approach is provided in Fig. 1. In summary, obtaining PAL with the proposed approach can be seen as a model- and score-agnostic way to detect candidate SNPs using neural networks trained for phenotype prediction. In the next section, we propose a method to estimate *P*-values for SNPs in PAL.

### Obtaining statistical significance

The main approach with the $\theta$ threshold weighting is designed for mitigating random effects of stochasticity (i.e. a few ‘rogue’ models presenting high-scoring non-associated loci), but it does not provide a statistical framework for controlling false positives (FPs). A common approach for this purpose would be a complete permutation-based method, but since this would be computationally infeasible in our case, we instead devised a method to obtain *P*-values based on the general assumption that higher attribution values are more likely to indicate a true signal.

Initially, we trained 10 models with permuted labels (i.e. shuffled binary disease status) and obtained null MAS for each, using the same steps as described previously. Since MAS is calculated as the absolute mean over multiple samples, a reasonable expectation (due to the central limit theorem) for the null distribution would be a folded normal distribution with $\mu =0$, which was approximately satisfied in IG null MAS at the tail region of interest (Supplementary Fig. S2). We further compared multiple distributions (half-normal, GPD, gamma, exponential, Weibull, and lognormal) at the tail region using Akaike information criterion and Bayesian information criterion, demonstrating further support for half-normal (Supplementary Fig. S3).

After deciding on the null distribution, we estimated the scale parameter $\sigma = 4.2\mathrm{e}{-6}$ from the pooled null MAS and sampled 10 different MAS vectors from the fitted half-normal distribution. To compute AMAS for both simulated and real datasets, we reordered each of the 10 sampled null MAS vectors to match the exact ranking of each of the observed 10 MAS vectors. This procedure emulates dependencies both within (i.e. due to LD and neural network connectivity) and between vectors (i.e. due to shared training data and architecture). We then averaged sampled null MAS vectors and calculated AMAS as previously described. This sampling procedure was repeated 100 times, and *P*-values were derived by counting the number of sampled AMAS values exceeding the target AMAS. The pseudocode for the method is provided in Algorithm 1.

**Table:** Algorithm 1 Deriving *P*-values for AMAS.

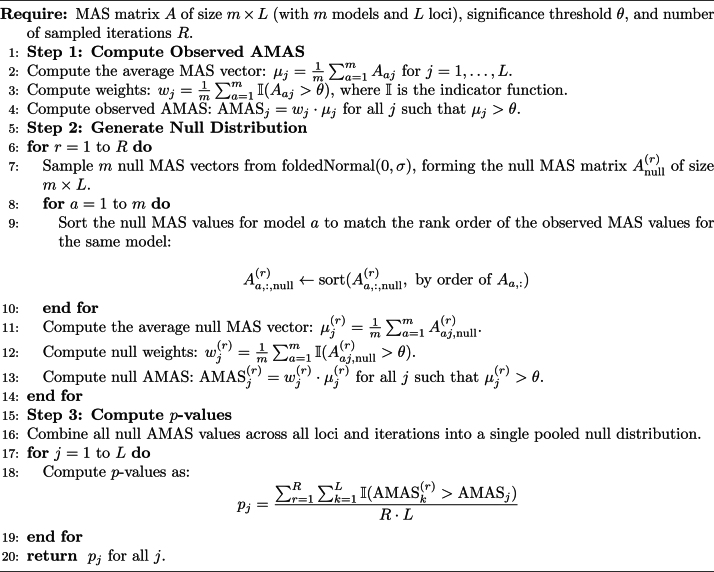

### Conventional GWAS

To compare our method to conventional linear GWAS, we initially performed logistic regression (LR) using all case (*n* = 1814) and control (*n* = 7325) samples (both in real and simulated scenarios) using the top 3 principal components (PCs) from principal component analysis as covariates. LR without PC covariates did not affect the results (as there is no or limited population structure in the EstBB dataset) (Supplementary Fig. S4). We used Python-3.9 and statsmodels 0.14.1 [33] for the LR code. Furthermore, we also performed an association analysis using a state-of-the-art LMM-based tool REGENIE v2.2.4 [3] on real and simulated data via the exact protocol described by Pujol Gualdo *et al.* [34], except for the filtering steps to retain the same individuals and genomic positions as LR.

### DeepCOMBI

We applied DeepCOMBI (DC) to the simulated phenotypes as described by Mieth *et al.* [12], using the available public code with minimal adaptations. Briefly, per chromosome, we converted biallelic SNP genotypes to the DC character matrix format and used the provided guidelines to train the Montaez-style dense neural network classifier on case–control labels (two hidden layers, L1/L2 regularization, dropout, Adam; hyperparameters as in the original paper’s ‘real data’ setting). After training, we applied the layer-wise relevance propagation implementation from the DC repository to obtain per-SNP relevance scores and selected the top-k SNPs per chromosome (*k* = 200, as used in the paper, and *k* = 10, for a stricter selection). For these DC-selected SNPs, we computed standard ${\chi }^2$ association *P*-values. Permutation-based approach proposed in the DC article to estimate statistical significance would be infeasible since we would have to obtain different thresholds for different chromosomes and perform permutations over six simulation scenarios. Therefore, we compared DC with other methods over fixed significance thresholds.

### Functional analyses

For the SCZ dataset, we defined the lowest *P*-value SNPs as lead SNPs and genes in the $\pm 100$ kb region of the detected SNPs in each PAL as positionally mapped genes. For expression analyses and eQTL mapping, we used the variant-gene association dataset (GTEx_Analysis_v8_eQTL) of the open access Adult Genotype Tissue Expression (GTEx) Project [35]. For all SNPs in PAL, we assessed the significance of expression levels (significance defined as pval_nominal < pval_nominal_threshold in significant variant-gene pairs dataset from GTEx *.signif_variant_gene_pairs.txt.gz files) in all brain tissues and reported eQTL-mapped genes for each PAL as genes for which the expression was altered significantly. To assess the functionality of positionally mapped genes in the detected PAL, we used the GENE2FUNC tool in FUMA [36], which leverages tissue-specific expression levels and phenotype enrichment based on the GWAS catalog [37]. Additionally, to assess the function of each variant, we used the g:Profiler g:SNPense tool [38].

To compare our findings for SCZ with the literature, we used LDtrait v6 [39]. For all detected SNPs in each PAL, we checked for ±500 kb region GWAS Catalog SNPs using $r^2 > 0.1$ threshold and European populations. In addition, to obtain a baseline probability of detecting SCZ- and BPD-associated SNPs by chance, we queried 100 random SNPs out of 290 522 from our SNP array framework using LDtrait with the same parameters.

### Computational Resources

The training of a single genome-wide model for 1000 epochs using an NVIDIA Tesla A-100 GPU with 40 GB vRAM lasts around 7–9 h, resulting in a total of 70–90 h for the training of 10 models for a single phenotype without parallelization. After training of 10 models for the target phenotype and null model training, the pipeline for obtaining PAL and *P*-values lasts around 5–20 h using the same GPU resource (to obtain feature importance scores), a single CPU core, and 64–256 GB RAM.

### Ethics statement

The activities of the EstBB are regulated by the Human Genes Research Act, which was adopted in 2000 specifically for the operations of the EstBB. Individual-level data analysis was carried out under ethical approval 1.1-12/624 (issued 24 March 2020), 1.1-12/2618 (issued 04 August 2022), and 1.1-12/3454 (issued 20 October 2022) from the Estonian Committee on Bioethics and Human Research (Estonian Ministry of Social Affairs), using data according to release application [6-7/GI/2577 V10] from the Estonian Biobank.

## Results

### Simulations

As post hoc approaches that can be applied to any trained neural network model, we evaluated the performance of the SM, IG, and PM-based feature importance methods across six different phenotype simulations, which varied by noise scaling factors ($k$ = 1, $k$ = 2, $k$ = 3) and the number of causal SNPs (100 and 1000). We designated a signal as true positive (TP) if one or more SNPs within a detected PAL block (i.e. blocks formed by clumping detected SNPs >100 kb distance) were in close proximity ($\pm 100$ kb, approximately $\pm 20$ SNPs) to a causal position. This approach both accounts for the difficulty of pinpointing the exact causal SNP due to LD between proximal SNPs and also mitigates bloated TP counts due to multiple signals from the same LD blocks. The results showed that the IG method excels over different simulation settings with the strict $\theta$ threshold in terms of controlling FPs (i.e. type I errors) compared with other methods and also performs fairly well in low and moderate noise settings with the relaxed $\theta$ threshold (Supplementary Figs S5 and S6).

**Figure 2.**
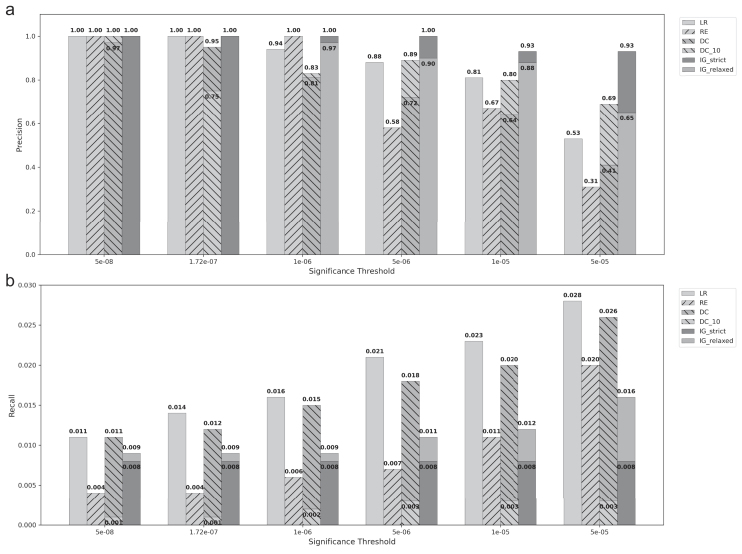
Precision (**a**) and recall (**b**) (*y*-axes) of LR, RE, DeepCOMBI (DC for *k* = 200 and DC_10 for *k* = 10), and integrated gradient (IG_strict and IG_relaxed, with strict and relaxed $\theta$ thresholds, respectively) methods over all simulation scenarios for different *P*-value significance thresholds (*x*-axes).

Since IG had better control of FPs and also satisfied the assumption of half-normal null distribution for obtaining *P*-values (see the ‘Materials and methods’ section and Supplementary Fig. S2), we compared IG to LR, REGENIE (RE), and DC under the same simulation settings for different significant *P*-value thresholds, in which 1.7e−07 is the stringent Bonferroni threshold obtained by dividing *P*-value = 0.05 by the total number of SNPs (Fig. 2 and Supplementary Figs S7 and S8). We performed comparisons for different *P*-value thresholds since multiple testing correction is not straightforward for neural network models (see the ‘Discussion’ section). The IG method overall had better precision and less FPs than other methods for most simulation parameters and significance thresholds, albeit generally presenting fewer TPs than LR and DC. All methods had low recall due to high false negatives, but DC (*k* = 200) and LR had the highest recall over different significance thresholds. We further compared the types of causal SNPs (dominant/recessive versus interactive) detected by these approaches (Supplementary Fig. S9). Interestingly, mainly positions with interactive genetic effects were detected, and there was no apparent difference between the IG, DC, and LR methods in this aspect, but RE detected more dominant/recessive effect SNPs. Finally, we visualized the performance of IG on Manhattan plots obtained from LR, demonstrating the capacity of both linear and nonlinear approaches for detecting multiple causal SNPs (Supplementary Fig. S10).

### Application to the EstBB SCZ cohort

After testing the approaches using simulations, we applied the IG method on the preliminary small T1D cohort and the full SCZ cohort from the EstBB to detect PAL. We opted not to use SM and PM on real data due to overall lower precision compared with IG and higher false positive rates under certain simulation scenarios (Supplementary Figs S5 and S6). For the T1D cohort, we were able to capture the highly associated *HLA* region signal despite the low sample size (Supplementary Fig. S11). However, we could not obtain well-calibrated *P*-values because the null distribution did not follow the expected half-normal (Supplementary Fig. S11c and d; see the ‘Discussion’ section).

**Figure 3.**
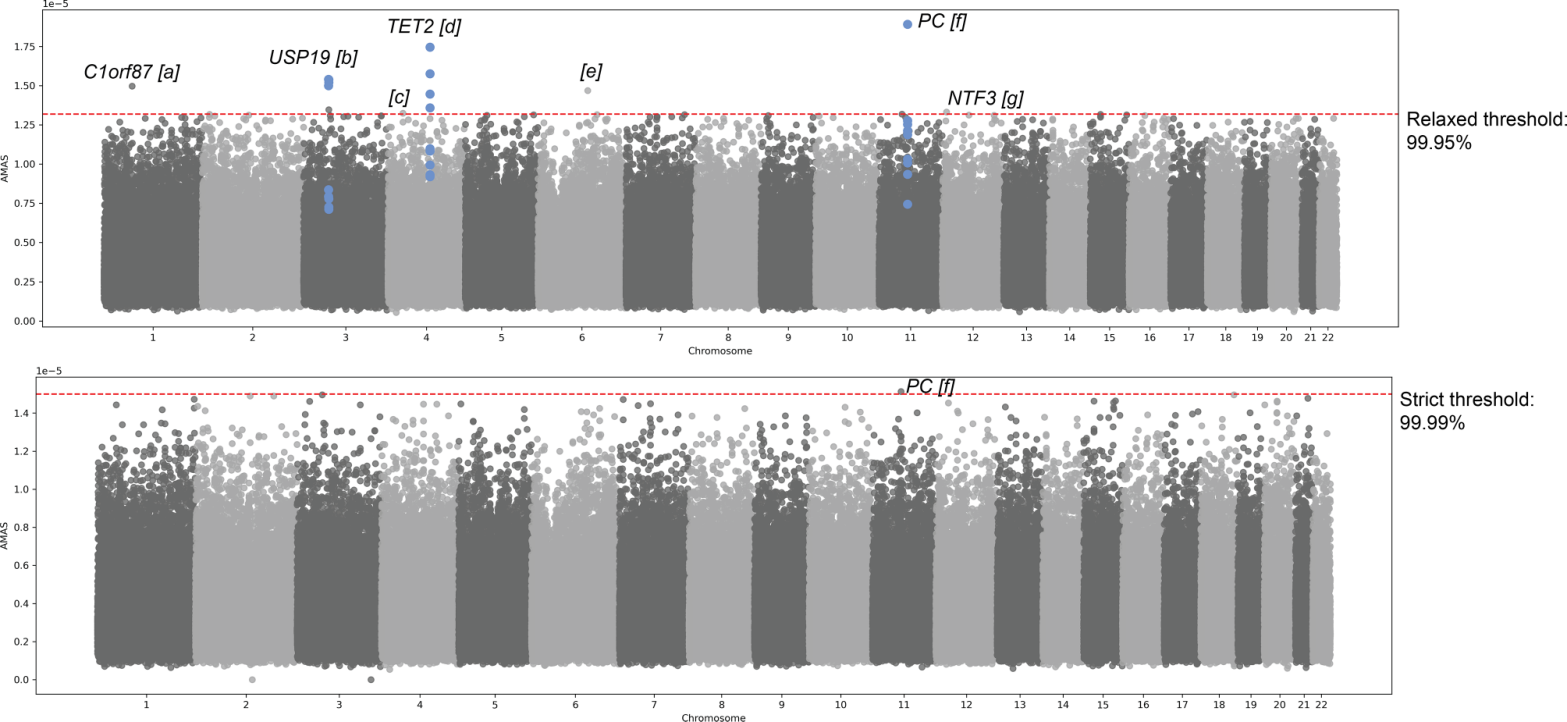
PAL detected by the IG approach. Red dashed lines indicate $\theta$ thresholds (relaxed and strict), and blue markers indicate PAL above threshold over all trained 10 models (i.e. $\mathrm{ PAL_{Common}}$). The closest protein coding gene to the lead SNP in $\pm 100$kb region was provided for PAL (a–g).

For the SCZ cohort, we detected a single locus (PAL f, total 1 SNP) with the strict $\theta$ threshold and multiple loci (PAL a–g, total 14 SNPs) with the relaxed $\theta$ threshold in genomic regions with multiple protein-coding genes (Fig. 3, Table 1, and Supplementary Table S2). The signal with the highest confidence (*P*-value ${\lessapprox} 5 \times 10^{-8}$) on chromosome 11 (PAL f) has been found to be associated with bipolar disorder (BPD, a genetically closely related trait with SCZ [40]), with the loci reported in literature including all the genes we report with positional and eQTL mapping [41]. The SNP rs7944999 in this region is in LD ($r^2 = 0.33$) with the SCZ-associated SNP rs1892928 within *SYT12* gene [19, 20]. Furthermore, it is also in LD ($r^2 = 0.32$) with rs10896135 located in *C11orf80*, a 98-kb open reading frame, and rs7122539 ($r^2 = 0.45$) in *PC*, which are similarly associated with BPD [41, 42]. Detected PAL b is a gene-dense region found to be associated with mood disturbance (depressive, psychotic, and manic symptoms; bipolar II; and major depressive disorder), with almost complete overlap with our positionally and eQTL-mapped genes [43]. This region includes rs4955417 ($r^2 = 0.57$ with PAL b SNP rs12631989), which has been associated with depressive symptoms and neuroticism and was mapped to *IHO1* and *C3orf62* [44, 45]), and rs7617480 ($r^2 = 0.46$ with PAL b SNP rs4955418), linked with major depressive disorder and unipolar depression, and mapped to *KLHDC8B* [46–48]. The PAL d region on chromosome 4 contains multiple variants linked to neuropsychiatric disorders. For instance, rs7674220 ($r^2 = 0.34$ with PAL d SNP rs2454206) has been associated with SCZ and cognition and mapped to *TET2* [49]. Additionally, rs10010325 (PAL d SNP) (mapped to *TET2*), rs2726528 ($r^2 = 0.57$), rs2713871 ($r^2 = 0.43$), and rs1603705 ($r^2 = 0.25$) (all mapped to *PPA2*), which have been reported to have pleiotropic effects for ADHD, autism spectrum disorder, and intelligence [50]. An interesting signal we detected is PAL g on chromosome 12, which we positionally mapped to *NTF3. NTF3* encodes neurotrophin-3 protein that has been shown to be linked to SCZ in multiple studies [51, 52], but with no reported SNP in GWAS Catalog for SCZ.

**Table 1.** Detected PAL with lead SNPs and mapped protein coding genes

PAL	Lead SNP	Chr:Pos	*P*-value	Positional mapped (±100 kb)	eQTL mapped
a	rs4915842*	chr1:59988397	$1.2 \times 10^{-5}$	C1orf87*, CYP2J2	NaN
b	rs6779394***^,$\dagger $^	chr3:49120338	$7.8 \times 10^{-6}$	C3orf62, C3orf84, CCDC71, DALRD3, GPX1, IHO1, IMPDH2, IP6K2, KLHDC8B, LAMB2, NDUFAF3, PRKAR2A, QARS1, QRICH1, RHOA, SLC25A20, USP19*, USP4	*AMT, CCDC71, DALRD3, GMPPB, GPX1, KLHDC8B, NCKIPSD, NICN1, P4HTM, QRICH1, WDR6*
c	rs12642383*	chr4:28192894	$3.5 \times 10^{-5}$	NaN	NaN
d	rs10461139**	chr4:105184142	$3.4 \times 10^{-6}$	PPA2, TET2*	NaN
e	rs7454792	chr6:82072955	$1.9 \times 10^{-5}$	*IBTK*	NaN
f	rs7944999**^,$\dagger$^	chr11:66920392	${\lessapprox }5 \times 10^{-8}$	C11orf80, C11orf86, LRFN4, PC*, RCE1, SYT12	*PC*
g	rs11063650^* ^	chr12:5403667	$3.4 \times 10^{-5}$	*NTF3*	NaN

Genes encompassing lead SNPs are marked with a star.

SCZ related SNPs in GWAS Catalog in ±500 kb region with *LD > 0, **LD > 0.1, ***LD > 0.5.

BPD related SNPs in GWAS Catalog in ±500 kb region with ^†^LD > 0, ^$\dagger\dagger$^LD > 0.1, ^$\dagger\dagger\dagger$^LD > 0.5.

Estimated probability of a random SNP being in LD > 0.1 with an SCZ-related SNP in GWAS Catalog is $\sim$0.05.

Estimated probability of a random SNP being in LD > 0.1 with a BPD-related SNP in GWAS Catalog is $\sim$0.02.

In the GWAS Catalog, we identified 4653 SCZ-relevant unique SNPs (including pleitropic effects), which can be structured into 1370 loci by connecting SNPs within ±500 kb distance. Considering all PAL with the relaxed threshold, we detected 6, 3, or 1 of these previously established loci, depending on the overlap rule (i.e. ±500 kb region and LD > 0, 0.1, or 0.5 between established and PAL SNPs).

In addition, we assessed the aggregate protein-coding genes we detected in terms of enrichment in phenotypes based on the GWAS Catalog (Supplementary Fig. S12). Lowest enrichment *P*-value phenotypes were brain morphology and volume, with multiple genes also linked with depressive symptoms, mood instability, and intelligence. Among detected variants, rs7647812 (*PRKAR2A*), rs6779394 (*USP19*), rs7944999 (*PC*), and rs10461139, rs10010325, rs6855629, and rs2454206 (*TET2*) are targets of nonsense-mediated mRNA decay, while rs4955418 (*CCDC71*) and rs2454206 (*TET2*) are missense variants. The extended information about detected PAL and SNPs is provided in Supplementary Table S2. Detailed queries for all detected SNPs against LD proxy SNPs from the literature (i.e. ±500 kb, $r^2 > 0.1$) can be found in Supplementary Table S3.

**Figure 4.**
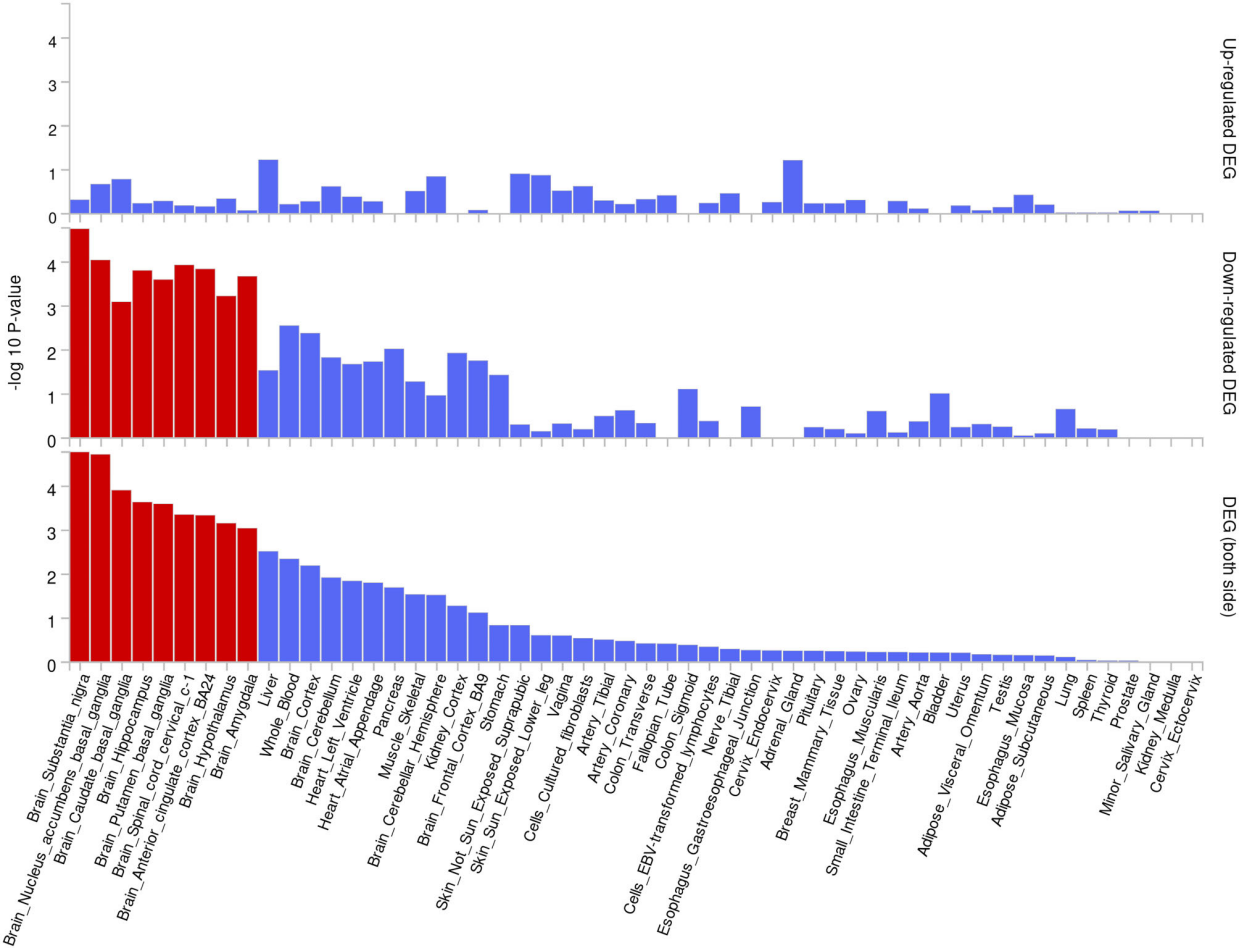
FUMA gene expression analysis of the detected genes for SCZ. Differentially expressed gene sets with significant enrichment based on Bonferroni correction are coloured in red, all of which belong to brain tissues.

We further assessed whether variants in detected PAL were enriched in genes expressed in brain. We initially obtained the empirical cumulative distribution for significant gene-tissue (only brain tissues) expression change in all 290 522 positions. The mean value for all positions was 0.858 (i.e. on average, an SNP had ${\lt}1$ gene–tissue combinations with significantly altered expression in brain), with most positions (250 692 SNPs) having no variants significantly affecting gene expression in brain tissues. However, the percentile rank for the detected PAL was 99.4, indicating potential enrichment in brain-expressed genes in these detected regions (Supplementary Fig. S13). As an additional step, we assessed expression specificity for the set of genes within the detected PAL and found significant downregulation in multiple brain tissues (Fig. 4).

Finally, we compared our approach to conventional GWAS using LR and REGENIE association analyses on the real dataset. Although there was a high correlation between MAS and −log(*P*) values (Supplementary Fig. S14), there was no significant signal detected using LR or REGENIE, and no overlap in signals at lower *P*-value significance thresholds (Supplementary Fig. S15). These conventional GWAS results are in line with findings from the UK Biobank, which used a similarly sized case sample set and reported no genome-wide significant hits [21].

## Discussion

### Method assessment and simulations

In this work, we assessed post hoc neural network interpretability methods for detecting trait-associated genomic loci and proposed a general framework for using these methods within a genotype–phenotype context. Simulations demonstrated that these approaches can yield low FP rates with a strict $\theta$ threshold, even in scenarios with relatively weak genetic signals. In addition, the method we proposed for obtaining *P*-values was robust not only to different scenarios but also to different $\theta$ thresholds for controlling FPs (Fig. 2 and Supplementary Figs S7 and S8). An important point to note is that the noise factor of 3 in the simulations corresponds approximately to the SNP-based heritability estimate for SCZ, which is around 0.25 [20]. However, this estimate is based on linear additive models, whereas our simulated traits were based solely on interactive and dominant/recessive effect positions, possibly making detection of associated loci substantially more difficult. Indeed, the neural network model was most successfully trained with the real dataset (i.e. smallest validation loss and least overfitting on average), which in addition increases confidence for the findings from the real dataset (Supplementary Fig. S16). As models demonstrated some overfitting on average for five out of the six simulation scenarios (Supplementary Fig. S16), performance metrics for our method can potentially be further improved by choosing the best models, using early stopping, more regularization, or simply better optimization. However, we refrained from such fine-tuning to demonstrate the robustness of the approach under suboptimal training outcomes.

This connects to a fundamental challenge in method development for detecting causal variants—the absence of ground truth of the underlying model for a trait (i.e. genetic architecture and the interactive landscape of genetic and environmental effects). Without knowing the underlying model, assessing these methods is challenging due to the likely discrepancy between simulated and real data domains. Another challenge for method assessment arises from LD. Even in simulations with ground truth information, evaluation metrics such as TP/FP counts can be substantially biased since methods (including LR) tend to produce highly correlated signals. We aimed to mitigate this with our definition of TP (i.e. if one or more SNP in a detected block is in $\pm 100$ kb region of a causal position), but methods with different capacities for detecting signals in LD regions might still be difficult to compare reliably. Future research could benefit from evaluation metrics better tailored for genomic data.

### Uncertainty quantification

An important obstacle to the broader adoption of the proposed approaches for GWAS lies in the difficulty of establishing reliable statistical significance measures. While our approach for obtaining *P*-values effectively controlled FPs in difficult simulation settings, a stable multiple testing correction remains a challenge due to complex dependencies stemming from the neural network architecture and gradient calculations. Furthermore, we used a pooled approach to obtain *P*-values based on a global null distribution for all features, essentially testing for relative extremity. This is fundamentally different from conventional GWAS, where each SNP is tested against its own null distribution. The global null approach allowed us to obtain meaningful *P*-values (i.e. can be corrected considering thousands of SNPs) via a feasible number of sampling iterations, but it also inherently limits the detection of small but significant effects. Another important limitation is that it might not be possible to estimate the null distribution for all types of feature importance scores or datasets (Supplementary Figs S2 and S11c and d), which is especially relevant if a complete permutation approach with multiple training runs is not computationally feasible, as in our application. However, even if a good null distribution cannot be estimated, PAL can still be obtained using a desired $\theta$ threshold to identify candidate SNPs as an exploratory analysis, as simulation results demonstrated that filtering based on $\theta$ limits FPs well (Supplementary Figs S5 and S6) and the highly associated *HLA* signal could be captured for T1D preliminary analysis without apparent false signals (Supplementary Fig. S11). As a conceptually different solution for controlling FPs, knockoff variables have been adopted in previous works [10, 13, 53]. Briefly, these are implanted ‘fake’ positions that mimic the distribution of real input features and enable the estimation of FP rates due to their independence from the output variable. However, sampling knockoff variables is not straightforward because deviations from the distribution of real input features would bias the false positive estimates. Furthermore, implementing a sufficient number of knockoff variables would potentially deteriorate the realness of data and significantly increase the computational burden due to the fully connected architecture of our model. Future parameter-light architectures (such as convolutions) or prior feature selection could be more suitable to explore this approach.

### Linear versus nonlinear models

Another important subject related to neural networks is model training. In our approach, we employed both heavy dropout regularization (including unusual dropout masking before the input layer) and ensemble-like multiple seed training to take advantage of stochasticity and limit FPs consequently. Interestingly, even with this heavy regularization, many models were trained successfully (i.e. positive correlation between training and validation loss), even though the predictive power of these models was limited. Overparameterization has been an important aspect for the success of deep learning in many domains [54], and our intuition on using a large model with regularization was based on this, since we are trying to model highly polygenic phenotypes potentially with some nonlinear structure. It is important to note that despite the high dropout masking before the first layer, the model still can see all inputs multiple times as the masking happens randomly for ~28 344 times over 1000 epochs with 256 batch size.

Despite that we did not optimize the models for prediction and predictive power is not the focus of this work, we still obtained the receiver operating characteristic curves for phenotype prediction in the real dataset, which were comparable between neural network, logistic regression, and REGENIE models, the last being slightly better (Supplementary Fig. S17). Initially, this might suggest that the real dataset primarily exhibits linear effects. Yet, as counter-evidence to the ‘mainly linear effects’ scheme, logistic regression or REGENIE could not detect any significant positions when applied to the real SCZ cohort, and there was no correspondence between the top detected positions of linear and neural network approaches (Supplementary Fig. S15), which might suggest the existence of nonlinearities in the real dataset. These aspects of effect types and how heavy regularization alters the capacity of neural networks for detecting nonlinearities are interesting subjects for future research. Considering the relatively small dataset for a case/cohort type analysis in this work, increasing the training data and experimenting with more simulation scenarios (such as including more nonlinearities, gene-environment interactions, or covariate effects) could be helpful to distinguish the differences between linear and nonlinear methodologies.

To be able to compare different methods fairly, we opted to use the same dataset with the non-imputed SNP array framework. Essentially, this reduces tagging and consequently discovery power. LR and REGENIE would detect more associations using the imputed framework with common variants, and the same could be true for the neural network-based approach since there would be more signal. However, training the models using the imputed framework with millions of SNPs would require very high computational resources, and training times might be completely infeasible. Another drawback would be the even higher feature size, exacerbating the so-called ‘curse of dimensionality’, which is not a problem for conventional GWAS because SNPs are tested separately.

### Interactive and dominant/recessive effects

One intriguing finding was that all approaches mainly detected positions with interactive effects in the simulation analyses. This may be due to dominant/recessive positions being more difficult to detect since the SNP effect completely disappears for a proportion of genotypes (see the ‘Materials and methods’ section). Additionally, the outcome space of functions defining complex trait genomics is considerably smaller than that of continuous variables, such as gene expression measurements, due to the ternary nature of genotype variables. Research into the topology of this space could provide valuable insights about the limitations of methods developed for *in silico* detection of causal loci and provide better roadmaps and improvement strategies.

### Application to real datasets

In the SCZ cohort application, we presented extensive evidence based on literature and enrichment analyses to demonstrate that the detected loci are related to neuropsychiatric disorders and brain function. Future research should focus on replicating these results for different populations, biobanks, and phenotypes. Furthermore, to mitigate confounding factors, we matched the distribution of case and control datasets based on age, sex, and BMI, and pruned the data to eliminate cryptic relatedness. Since EstBB dataset does not exhibit high population structure (e.g. LR findings did not change when PCs are used as covariates, Supplementary Fig. S4), we did not include PCs as input features for the neural network model. Future models could integrate potential confounding variables to improve generalizability over different datasets and reduce data preprocessing steps.

### Final remarks

Despite the state-of-the-art deep learning algorithms and the ever-increasing amount of data in biobanks, several challenges remain in the widespread adoption of neural networks for GWAS. These include stochasticity stemming from initial random states, lack of established uncertainty quantification, and the absence of ground truth for the underlying genetic and environmental landscape of complex traits. These challenges are fundamentally connected to intriguing questions, such as the contribution of nonlinear effects (gene–gene or gene–environment interactions, or other higher-order polynomial forms) or omnigenic versus polygenic models for complex disease development [55]. In the near future, assumption-free interpretable neural networks may become essential tools for exploring these questions and could ultimately replace linear models as the standard approach for GWAS and for generating interpretable polygenic risk scores.

## Supplementary Material

lqag019_Supplemental_Files

## Data Availability

Relevant code for the neural network model, interpretability methods, and logistic regression with mock simulated data can be found at https://github.com/genodeco/Interpreting-ANNs-for-GWAS and https://doi.org/10.5281/zenodo.18389067. Access to the Estonian Biobank datasets is subject to an application process requiring relevant ethical clearances. The detailed application procedure can be found at https://genomics.ut.ee/en/content/estonian-biobank.
