# Supplementary material for: Interpreting artificial neural networks to detect genome-wide association signals for complex traits
Source: NAR Genom Bioinform. 2026 Feb 23;8(1):lqag019. doi: 10.1093/nargab/lqag019 (PMC12964191; doi:10.1093/nargab/lqag019)
Supplement: lqag019_Supplemental_Files [file lqag019_Supplemental_Files.zip › supplementary_yelmen_interpreting2026.pdf]

Supplementary Figures

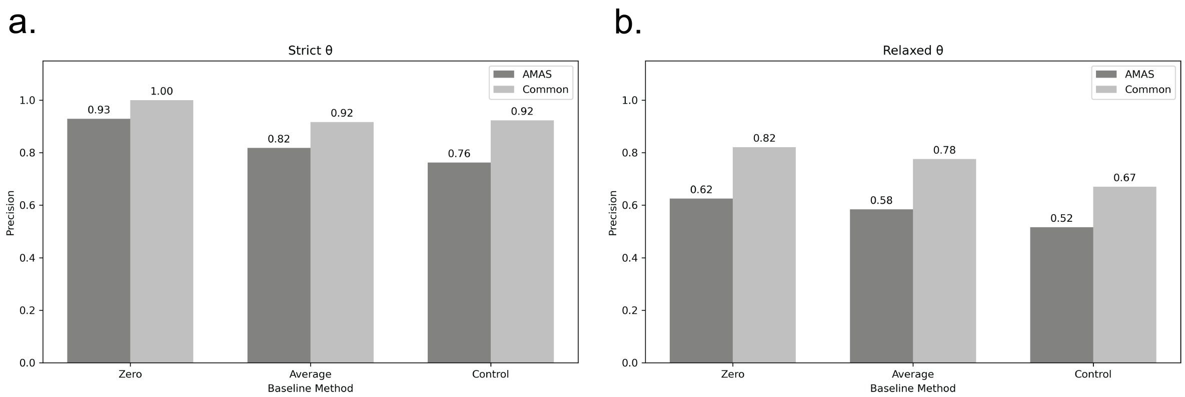

S1 Fig. Assessment of different baselines (i.e., zero, average allele dosage and control genotypes) for the integrated gradient (IG) method with strict (**a**) and relaxed (**b**) θ thresholds based on precision over all simulation scenarios.

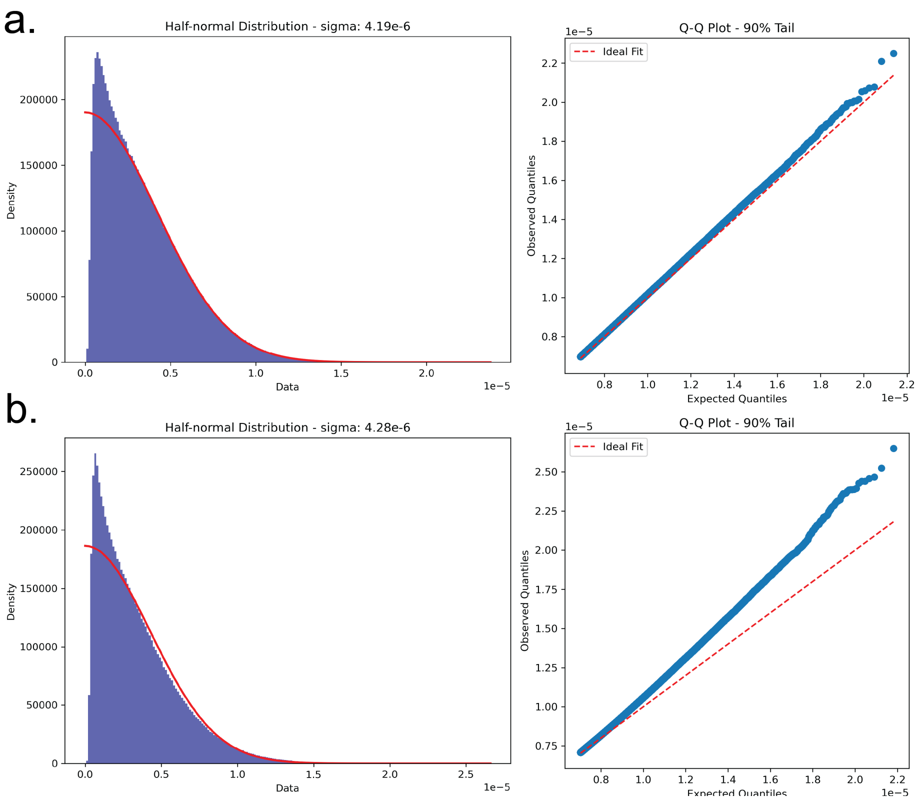

S2 Fig. Assessment of half-normal distribution fit to **a)** integrated gradient (IG) and **b)** saliency map (SM) null mean attribution score (MAS) distribution obtained via 10 model training with permuted labels. Red lines indicate the theoretical fit. Right column is the Q-Q plot of expected versus observed values for the 90th percentile tail.

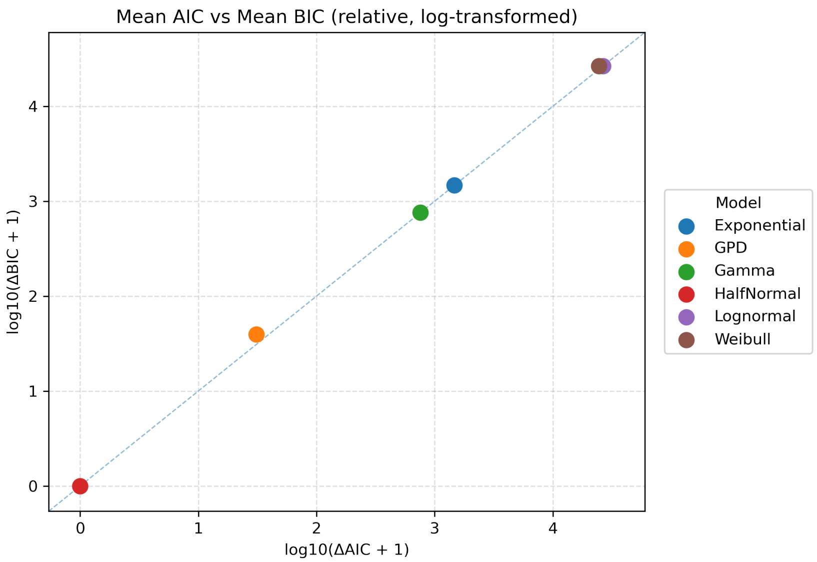

S3 Fig. Mean AIC and BIC values for six candidate tail models, expressed as log-transformed differences ($\log_{10}(\Delta + 1)$) from the best-performing model. Values were averaged over four tail cutoffs (90, 95, 99, 99.9 percentiles). Lower values indicate superior support where (0,0) coordinate indicates the best model.

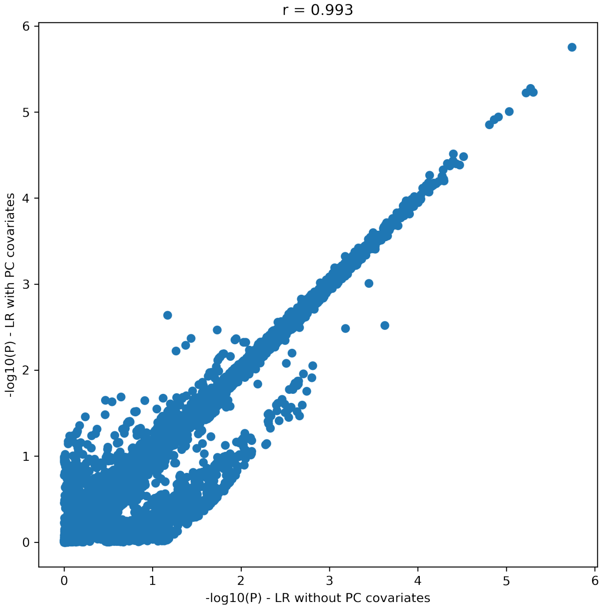

S4 Fig. Comparison of logistic regression (LR) p-values without (x-axis) and with (y-axis) PC covariates.

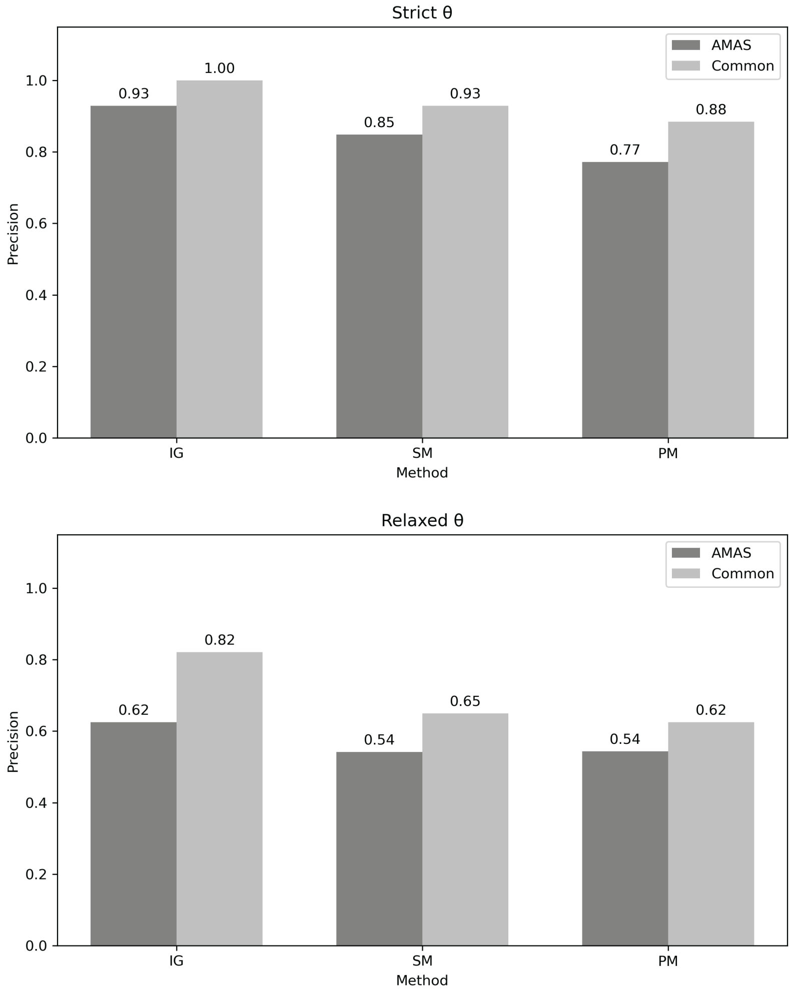

S5 Fig. Precision of different methods (IG: integrated gradients, SM: saliency map, PM: permutation-based) over all simulation scenarios with strict (above) and relaxed (below) θ thresholds.

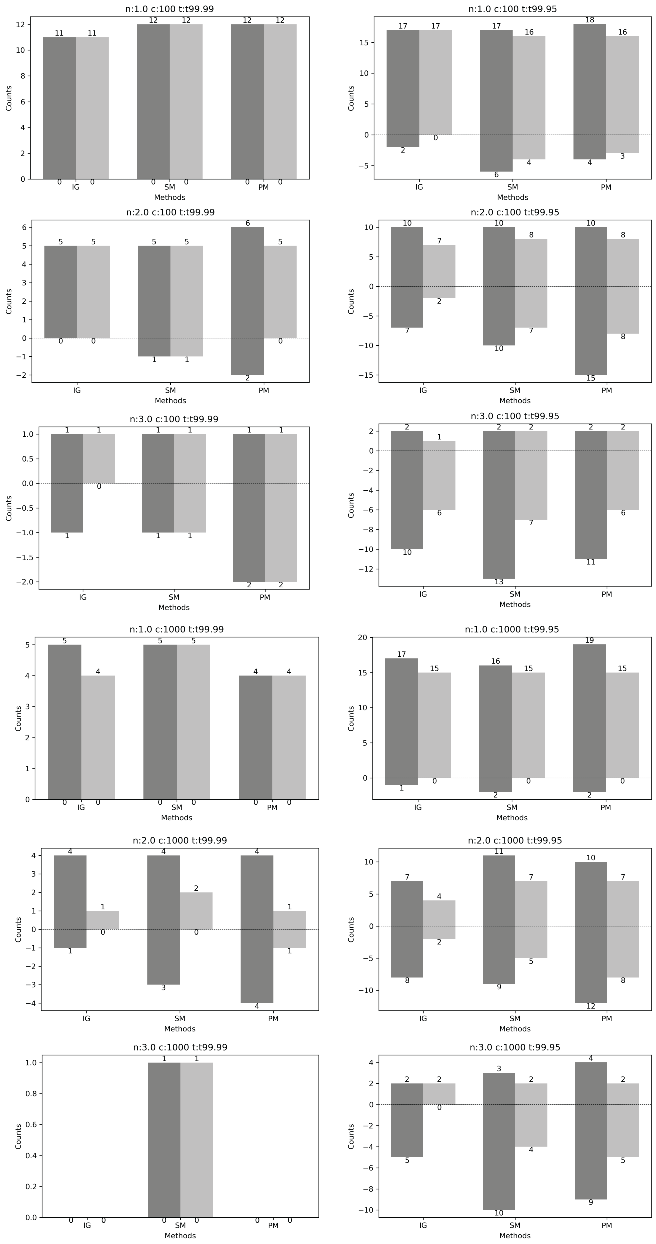

S6 Fig. Comparison of true positive (TP) and false positive (FP) counts for different methods based on various simulation scenarios and thresholds (n: noise factor, c: number of causal positions, t: θ threshold). A signal was determined to be TP if one or more SNP in a detected PAL block (i.e., blocks formed by clumping detected SNPs less than 100kb distance) is in close proximity (±100kb, approximately ±20 SNPs) with a causal position. Positive values (above 0 on the y-axis) indicate TP counts whereas negative values (below 0 on the y-axis) indicate FP counts. Methods defined in x-axis are integrated gradients (IG), saliency map (SM) and permutation-based (PM) approaches. First bars (dark grey) show TP/FP counts for PAL_{AMAS} whereas second bars (light grey) show TP/FP counts for PAL_{Common}.

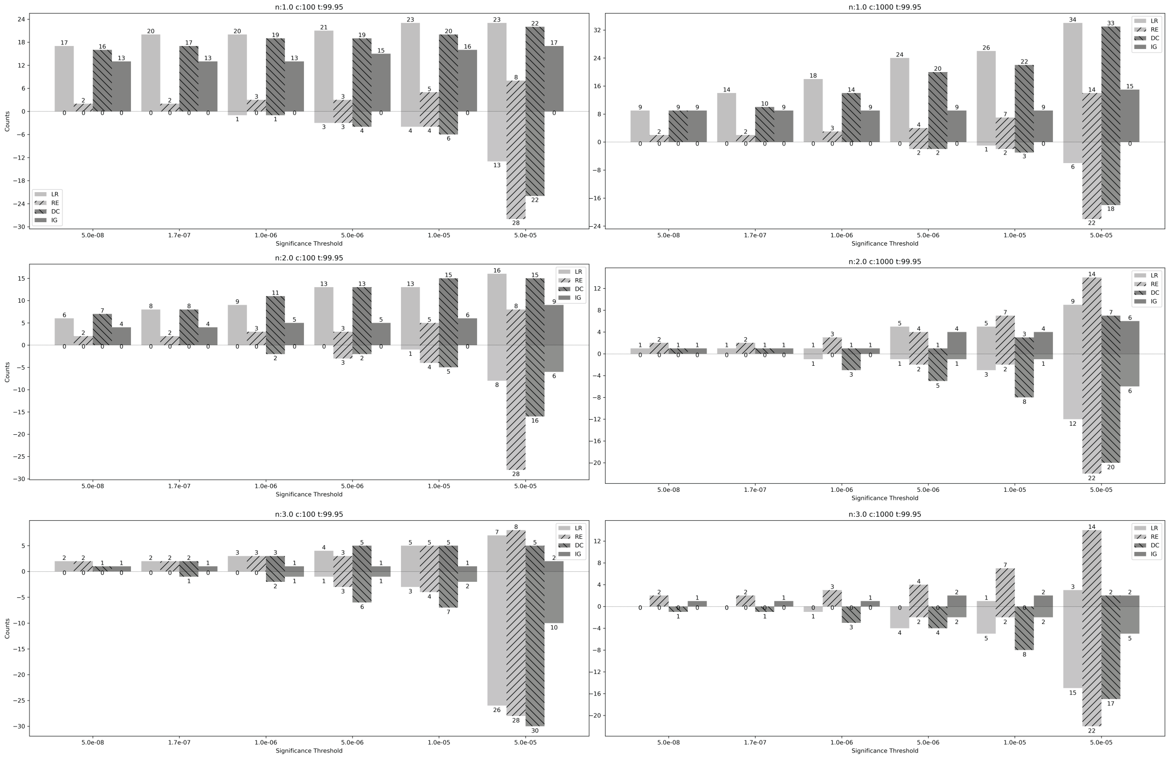

S7 Fig. Comparison of true positive (TP) and false positive (FP) counts for the logistic regression (LR), REGENIE (RE), DeepCOMBI (DC) and integrated gradient (IG) methods based on various simulation scenarios (n: noise factor, c: number of causal positions, t: relaxed θ threshold) and different significant p-value thresholds. A signal was determined to be TP if one or more SNP in a detected PAL block (i.e., blocks formed by clumping detected SNPs less than 100kb distance) was in close proximity ($\pm$100kb, approximately $\pm$20 SNPs) with a causal position. Positive values (above 0 on the y-axis) indicate TP counts whereas negative values (below 0 on the y-axis) indicate FP counts.

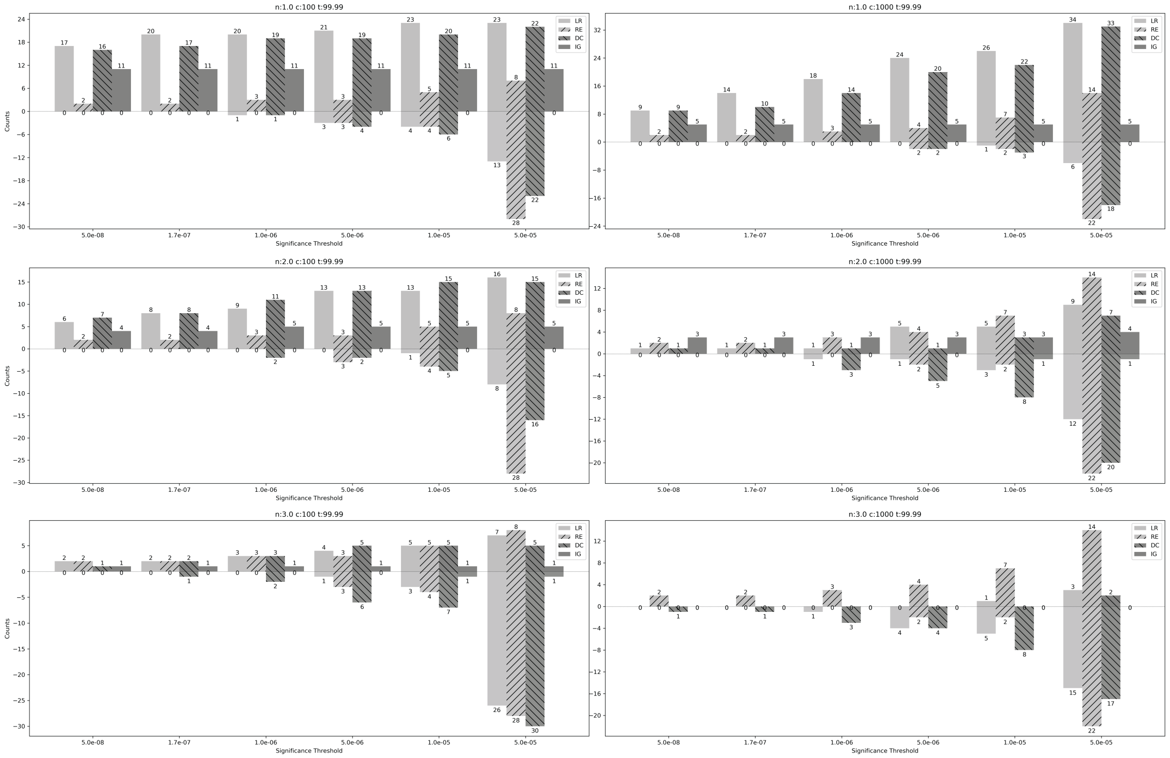

S8 Fig. Comparison of true positive (TP) and false positive (FP) counts for the logistic regression (LR), REGENIE (RE), DeepCOMBI (DC) and integrated gradient (IG) methods based on various simulation scenarios (n: noise factor, c: number of causal positions, t: strict θ threshold) and different significant p-value thresholds. A signal was determined to be TP if one or more SNP in a detected PAL block (i.e., blocks formed by clumping detected SNPs less than 100kb distance) is in close proximity ($\pm$100kb, approximately $\pm$20 SNPs) with a causal position. Positive values (above 0 on the y-axis) indicate TP counts whereas negative values (below 0 on the y-axis) indicate FP counts.

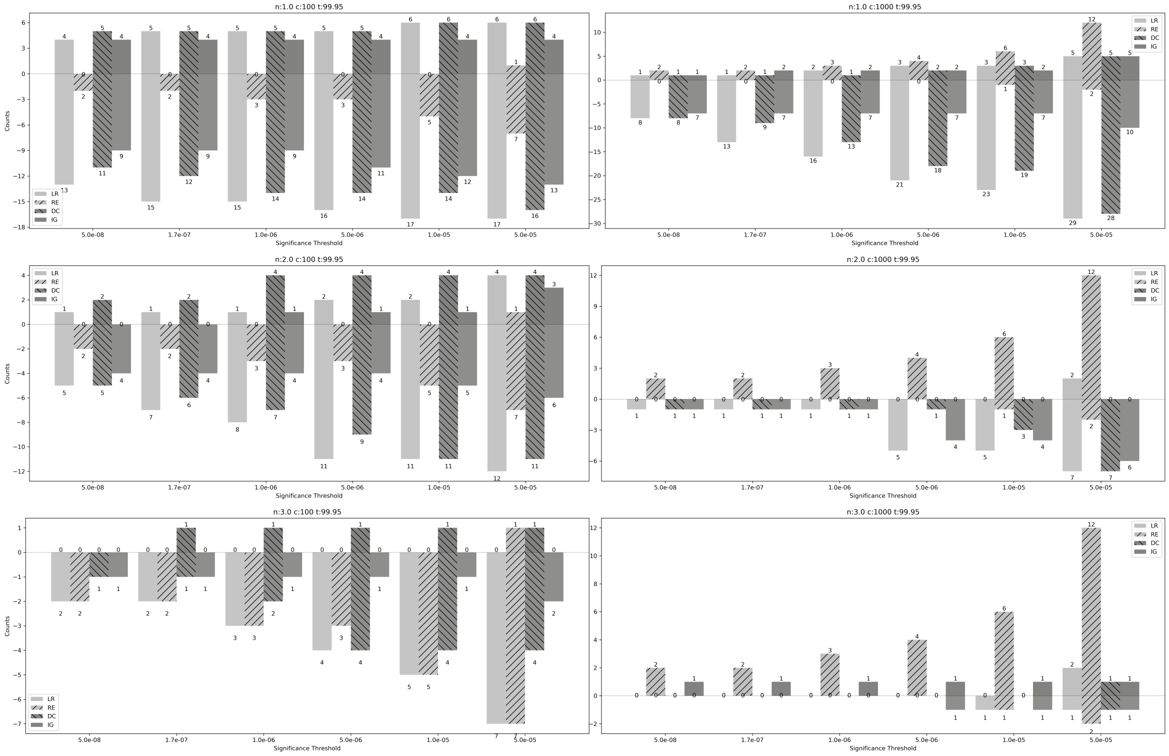

S9 Fig. Comparison of dominant/recessive (positive values) and interactive effect (negative values) position counts in correctly detected (true positive) positions for the logistic regression (LR), REGENIE (RE), DeepCOMBI (DC) and integrated gradient (IG) methods based on various simulation scenarios (n: noise factor, c: number of causal positions, t: relaxed θ threshold) and different significant p-value thresholds.

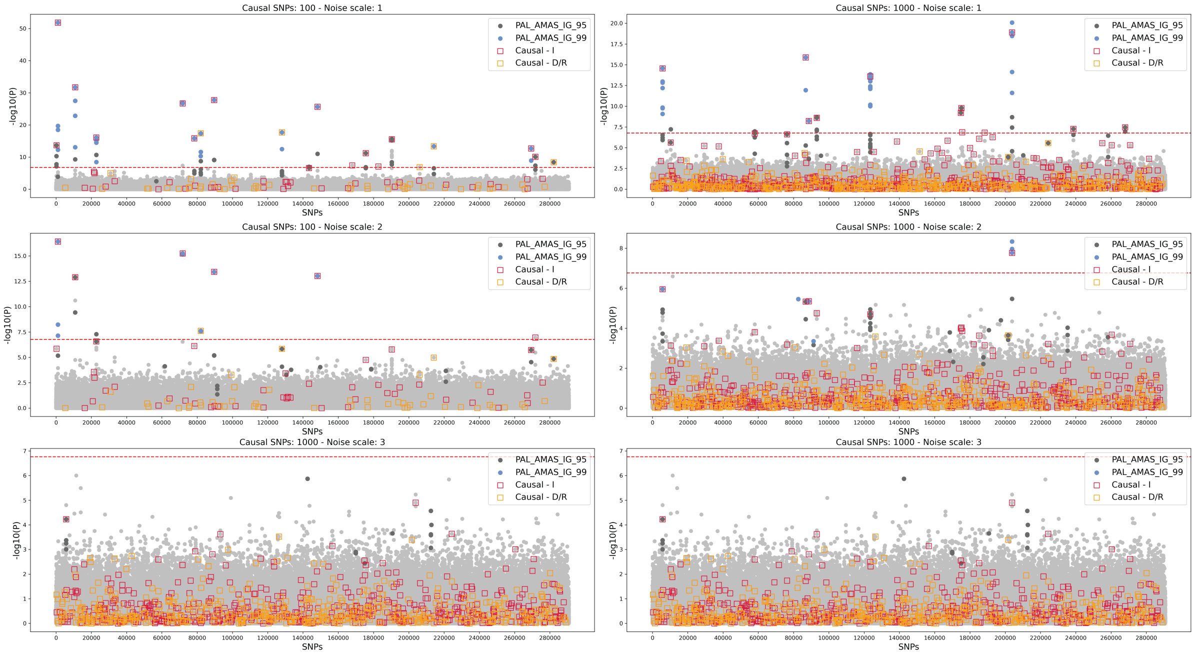

S10 Fig. Manhattan plots from LR with annotated PAL_{AMAS} detected via IG approach. x-axis denotes SNP positions over the whole genome, and y-axis denotes -log(P) values of the LR analysis. Each figure is a different simulation scenario with varying amounts of noise and causal SNPs, as described in the subcaptions. Dashed red lines denote the significance threshold for LR (with Bonferroni corrected p-value = 0.05 divided by the number of tests). Red squares denote causal SNPs with interactive effects (Causal - I), and orange squares denote causal SNPs with dominant/recessive effects (Causal - D/R). Blue and black dots represent PAL_{AMAS} detected with strict (99.99 percentile) and relaxed (99.95 percentile) θ thresholds, respectively.

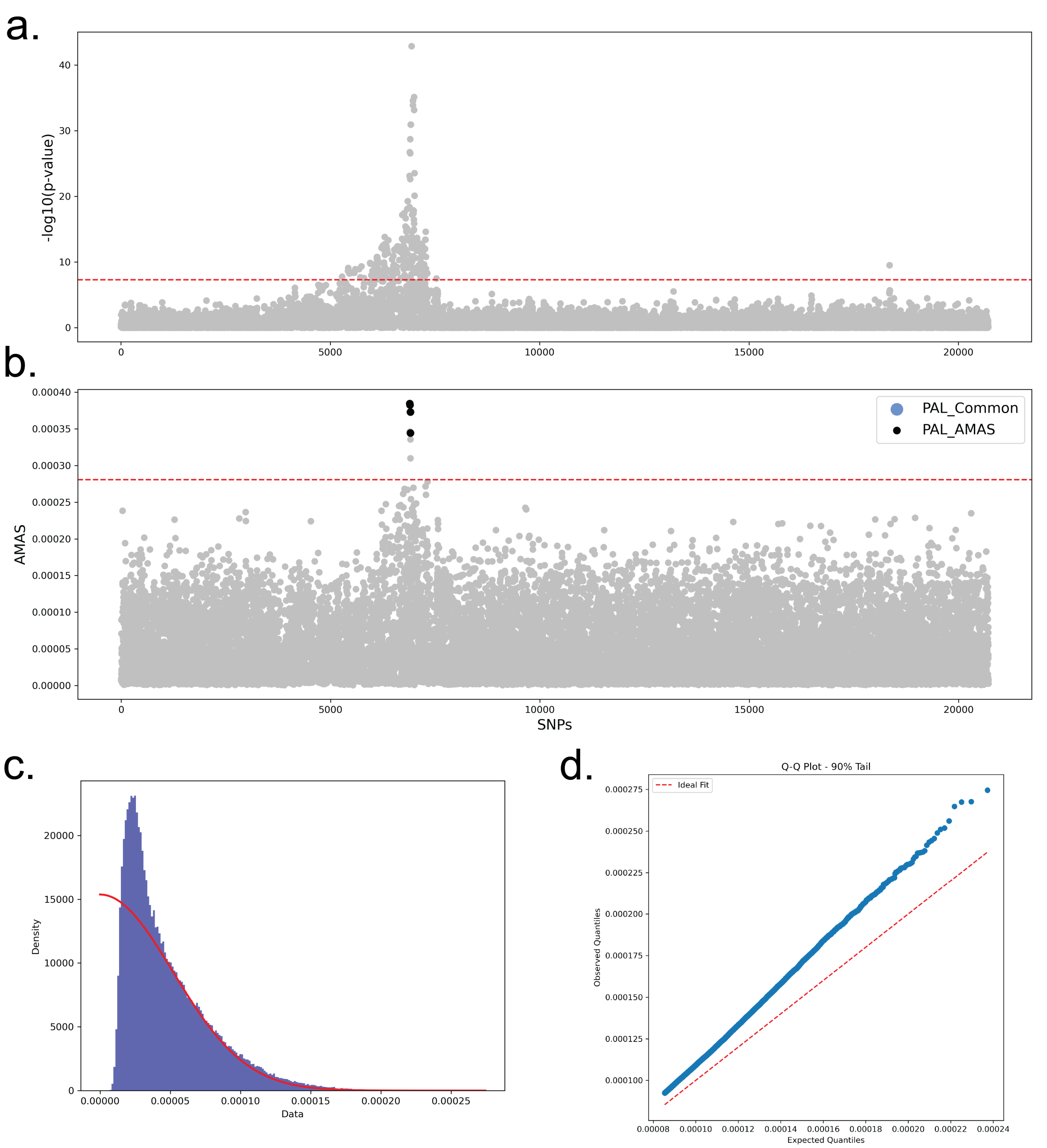

S11 Fig. Results of the preliminary analysis with type 1 diabetes cohort (552 case, 1104 control samples with 20,714 SNPs from chromosome 6). **a**) Logistic regression -log(P) values (y-axis) for each SNP (x-axis). **b**) Adjusted mean attribution score (AMAS) (y-axis) for each SNP (x-axis) from the proposed integrated gradient (IG) method. **c**) Assessment of half-normal distribution fit for IG null mean attribution score (MAS) distribution obtained via 10 model training with permuted labels. **d**) Q-Q plot of expected versus observed values for the 90th percentile tail.

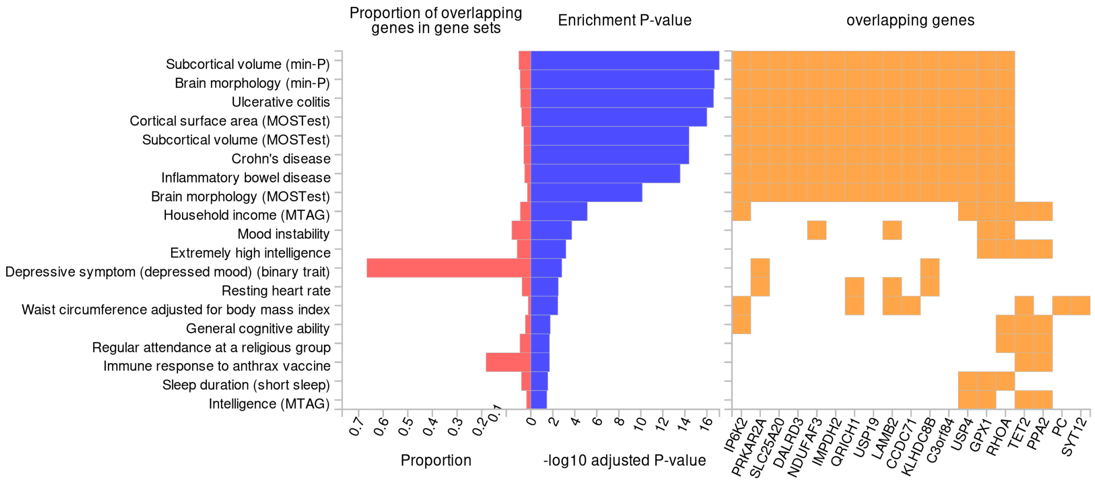

S12 Fig. FUMA functional analysis of detected genes based on enrichment in GWAS-catalogue gene sets.

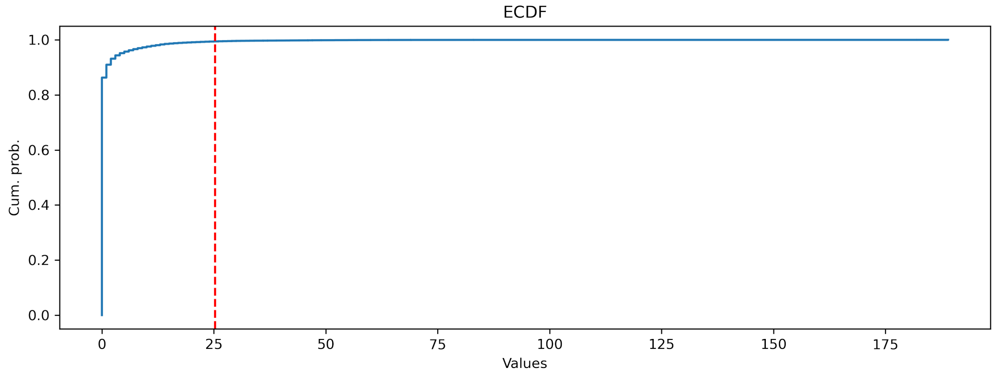

S13 Fig. Empirical cumulative distribution function (ECDF) plot of brain tissue expression for all 290,522 SNPs. Values (x-axis) correspond to the number of gene-tissue (consisting of all types of brain tissues) combinations with significant expression change. Percentile rank for the average value over detected PAL set is 99.4 (354 significant gene-tissue expression for 14 detected SNPs).

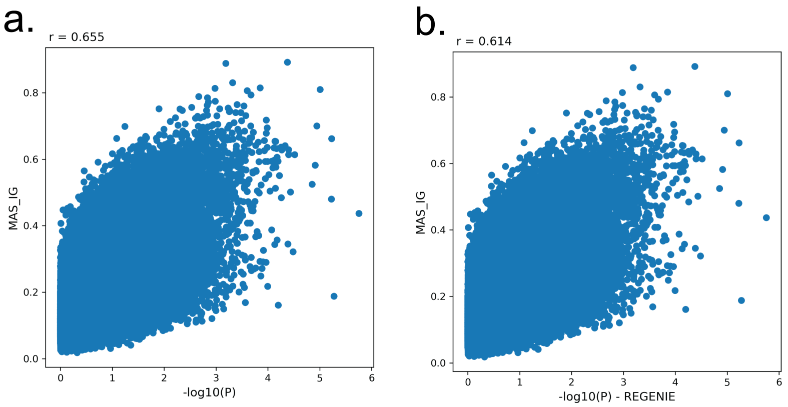

S14 Fig. Correlation between mean attribution score (averaged over 10 different models) obtained via integrated gradient (MAS_IG) approach and -log(P) values obtained via **a)** logistic regression and **b)** REGENIE association analysis using SCZ dataset. Provided r values are Pearson's correlation coefficient.

a.

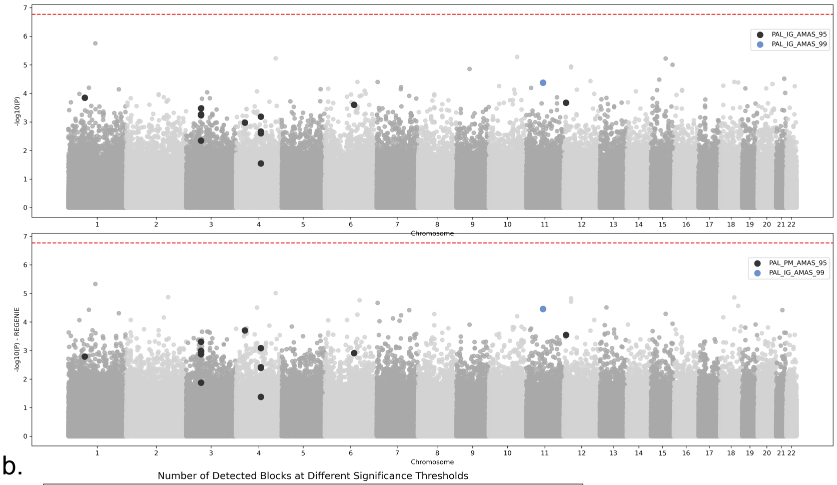

b.

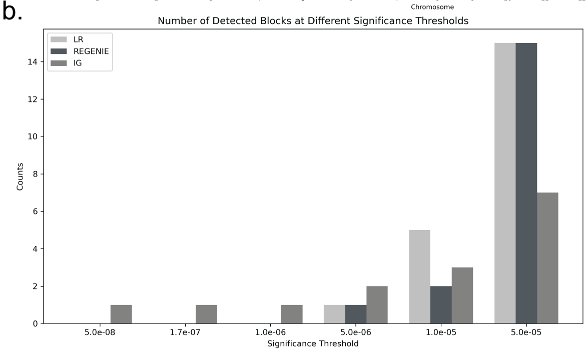

S15 Fig. **a)** Manhattan plots from LR (top) and REGENIE (bottom) association analysis with annotated PAL_{AMAS} detected via IG approach for SCZ dataset. Blue and black dots represent PAL_{AMAS} detected with strict (99.99 percentile) and relaxed (99.95 percentile) θ thresholds, respectively. Red dashed lines show Bonferroni significance thresholds for LR and REGENIE analyses. **b)** Number of detected blocks for IG, LR and REGENIE methods under different p-value significance thresholds. There was no common signal between IG and LR/REGENIE in any p-value threshold, whereas all signals detected by REGENIE were also detected by LR with 1e-05 and 5e-06 thresholds.

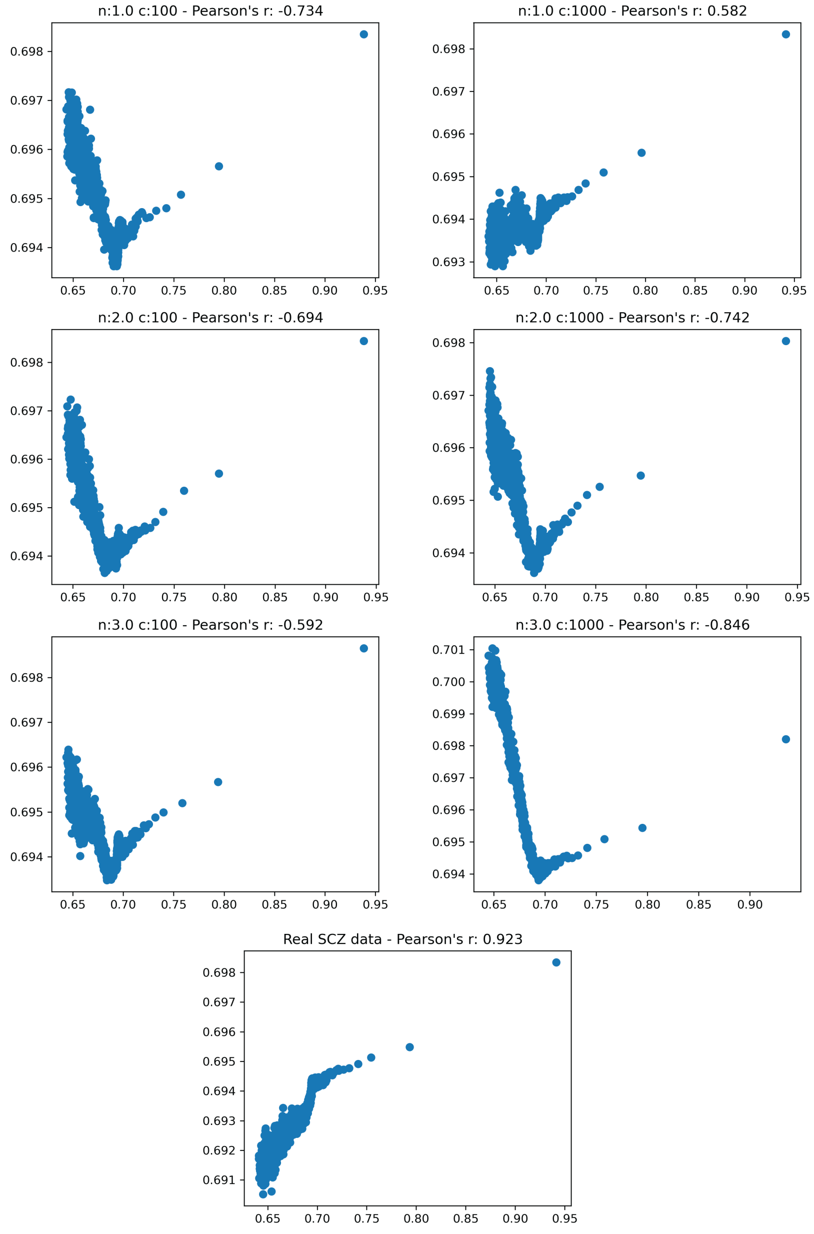

S16 Fig. Comparison of neural network model training using simulated and real data, based on the correlation between training loss (x-axis) and validation loss (y-axis). Training and validation losses were averaged over 10 different models. In each plot, the rightmost point corresponds to average loss after first epoch and leftmost point corresponds to average loss at the end of training (1000th epoch).

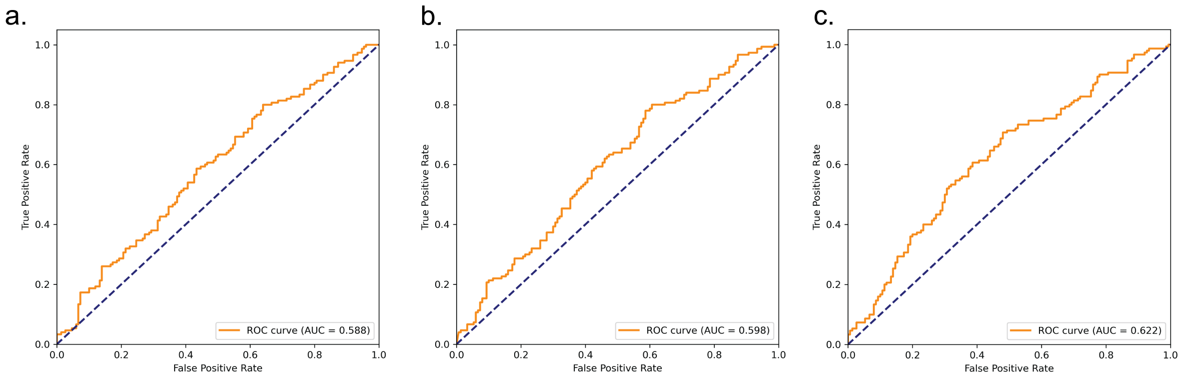

S17 Fig. Receiver operating characteristic (ROC) curves and area under the ROC curve (AUC) for **a)** neural network models trained for 1000 epochs (predictions averaged over 10 models trained with different seeds), **b)** logistic regression and **c)** REGENIE phenotype predictions. Predictions were performed on the same test genotypes (150 cases, 150 controls) not utilised in fitting or training for logistic regression and neural networks. For REGENIE, polygenic risk score prediction output was used.

Supplementary Table Legends

S1 Table. Detailed information on SNPs used for simulating phenotypes.
S2 Table. Detected PAL including all SNPs with mapped genes.
S3 Table. Detailed queries against GWAS Catalog for all SNPs in each PAL. LDtrait query was performed for ±500 kb region with $r^2 > 0.1$ threshold on European populations. Only phenotypes including at least one of the following strings is reported: "schizophrenia", "bipolar", "cognitive", "attention", "depression", "mood", "brain", "anxiety", "neuro". Schizophrenia and bipolar disorder related associations (including pleiotropy) are indicated with bold text.
